# Sequential Extraction and Attenuated Total Reflection–Fourier Transform Infrared Spectroscopy Monitoring in the Biorefining of Brewer’s Spent Grain

**DOI:** 10.3390/molecules28247992

**Published:** 2023-12-07

**Authors:** Ilary Belardi, Assunta Marrocchi, Vincenzo Alfeo, Valeria Sileoni, Giovanni De Francesco, Marco Paolantoni, Ombretta Marconi

**Affiliations:** 1 Department of Agricultural, Food and Environmental Sciences, University of Perugia, 06121 Perugia, Italy; ilary.belardi@studenti.unipg.it (I.B.); giovanni.defrancesco@unipg.it (G.D.F.); 2 Department of Chemistry, Biology and Biotechnology, University of Perugia, 06123 Perugia, Italy; assunta.marrocchi@unipg.it (A.M.); marco.paolantoni@unipg.it (M.P.); 3 Italian Brewing Research Centre (CERB), University of Perugia, 06126 Perugia, Italy; vincenzo.alfeo@unipg.it; 4 Department of Economic and Legal Sciences, Universitas Mercatorum, 00186 Rome, Italy; valeria.sileoni@unimercatorum.it

**Keywords:** brewer’s spent grain, biorefinery, biomass valorization, ATR-FTIR spectroscopy

## Abstract

The brewing industry plays a significant role in producing a substantial annual volume of by-products, which contributes to the global accumulation of food waste. The primary by-product generated is brewer’s spent grain (BSG), a lignocellulosic biomass rich in proteins, fiber, and moisture content. Leveraging biorefining and valorization techniques for BSG represents a promising strategy to enhance sustainability, resilience, and circularity within the brewing chain. To date, most studies have focused on extracting proteins from BSG. Yet, it is crucial to note that the fiber part of BSG also holds considerable potential for biorefining processes. This study introduces a novel sequential extraction method designed to integrally recover the major components of BSG. Notably, it introduces a reactive extraction approach that enables the simultaneous extraction and tuneable functionalization of the hemicellulose component. Additionally, the study assesses the utility of the attenuated total reflection–Fourier transform infrared (ATR-FTIR) spectroscopy as a user-friendly tool to monitor and evaluate the effectiveness of the fractionation process. This spectroscopic technique can provide valuable insights into the changes and composition of BSG throughout the extraction process.

## 1. Introduction

The ongoing globalization of markets is worsening the issue of food loss and waste throughout the entire food supply chain. According to the United Nations Food and Agriculture Organization (FAO), ~30–40% of the world’s annual food production is lost, significantly impacting global food security, the economy, and the environment. In 2019, worldwide food losses and waste were estimated to be around 1.6 billion tons [1,2], costing about EUR 2.3 trillion [3]. Furthermore, these losses contribute to 8–10% of the increase in greenhouse gas emissions, equivalent to about 3.3 gigatons of CO_2_ per year, due to disposal in landfills [1,2]. To address this issue, Sustainable Development Goal (SDG) 12, Target 12.3 of the “2030 Agenda for Sustainable Development”, adopted by the United Nations in 2015, aims to reduce global food losses and waste per capita by 50% [2]. Among various industries, the beverage sector significantly contributes to waste streams, accounting for approximately 26% of total food losses and waste [4]. Beer, one of the most popular alcoholic beverages worldwide, had a global production of ~1.86 billion hL in 2021 [5], with Europe contributing ~396 million hL [6]. Based on 2021 data from the European Beer Association, the EU is projected to produce about 425 million hL of beer by 2030 [6].

In the brewing process, three major by-products are generated: brewer’s spent grain (BSG), spent hop, and spent yeast. BSG accounts for ~85% of the total by-products [7], meaning that for every 100 L of beer produced, nearly 20 kg of wet BSG is generated as a by-product. This results in a massive global BSG production of ~40 million tons/year, with Europe alone producing ~8 million tons/year. In 2021, global BSG production was estimated at ~37.2 million, with ~7.9 million tons in Europe and ~340 thousand tons in Italy [6,8,9].

BSG is obtained at the end of the mashing process from the lautering stage and it consists of the insoluble component of the wort, including the seed coat, pericarp, and husk layers of barley grains. BSG is mainly composed of hemicelluloses (~25%, on a dry weight basis), cellulose (~17%), lignin (~28%), and proteins (~30%). The primary hemicelluloses in BSG are arabinoxylans (AX), which form a linear chain backbone of ß-(1→4)-D-xylopyranosyl residues, mainly substituted with α-L-arabinofuranosyl residues at O-2, O-3, and/or at O-2,3, though other positions may also have linkages and other substituents may be present. BSG has a high moisture content, ranging from 80% to 85%, making it prone to microbial growth and spoilage within a relatively short period, typically around 8–10 days. The chemical composition of BSG may vary due to factors like the type of grains used, harvest time, geographical location, malting and mashing conditions, and grain quality [7]. Despite variations, BSG’s composition, abundance, and environmentally friendly characteristics make it an attractive choice for circular bioeconomy initiatives. Yet, its high moisture content requires an additional and costly drying process to extend its shelf life. This drying, coupled with transportation costs, poses significant challenges to its efficient utilization. Furthermore, since BSG is mostly made up of lignocellulosic material (vide supra), it shows recalcitrance, making it resistant to structural disruption. Overcoming this recalcitrance is crucial to finding effective methods to maximize the value and utility of BSG in various applications within the bioeconomy framework.

Currently, within the EU, ~70% of BSG is used for animal feed, ~10% for biogas production, and ~20% is sent to landfills, with the latter resulting in an impact of ~513 kg CO_2_ equivalent/ton. Additionally, 5–10% of BSG is used in food production and fertilizer in agriculture [10]. Various attempts have been made to maximize the value of BSG by producing bioethanol, activated carbon, and adsorbents, substituting it for sawdust in brick making, and incorporating it into paper manufacturing [11]. BSG has also been explored as a filler in polymer matrices, although this often resulted in decreased mechanical properties [12].

To fully capitalize on the potential of BSG, it is essential to deconstruct, separate, and recover its valuable components. Efficient fractionation processes are necessary to isolate the primary constituents of the raw material for successful conversion into a range of value-added products. However, existing studies have mainly focused on recovering the protein fraction of BSG, driven by the increased demand for proteins in the food and feed sectors [13,14,15]. These studies face challenges, including high drying costs, expensive enzymatic species, and low efficiency in protein separation. The recovery of BSG’s fibrous part, including its structural components, has received less attention, due to the mentioned recalcitrance of the lignocellulosic structure. While some studies target the partial recovery of fibrous components alongside proteins, the separation processes often require the preliminary drying of BSG [16,17] and the use of costly solvents like deep eutectic solvents (DESs) [18] or ionic liquids (ILs) [19,20], which may present toxicity issues [21]. Also, the recovery of polysaccharides, particularly hemicellulose, typically occurs as hydrolysate, and obtaining it as a macromolecule could significantly enhance its value for biorefineries [12]. There is clearly a gap in the development of sustainable methods to fully fractionate and isolate BSG major components, and in the establishment of a cascade utilization of BSG based on its composition to produce value-added products.

In this study, we have developed an innovative sequential extraction process to recover the major components of BSG, offering significant advantages over the existing protocols. First, the process stands out by directly utilizing wet BSG, eliminating the need for costly and energy-intensive preliminary drying steps. Next, it employs a green extraction medium and operates under mild conditions. Additionally, a reactive extraction step has been incorporated, allowing for the simultaneous extraction and tuneable functionalization of hemicellulose. This unique feature enhances the value of hemicellulose for biorefineries, enabling the production of tailored derivatives for various applications. In addition, there is a need for fast and precise monitoring methods during the fractionation processes. While sporadic attempts have been made using techniques such as near-infrared spectroscopy (NIR), thermogravimetric analysis, and Raman scattering microscopy [22], these methods may lack user-friendliness or the desired accuracy. In this study, the use of attenuated total reflection–Fourier transform infrared (ATR-FTIR) spectroscopy is pioneered for fast process monitoring and analysis. ATR-FTIR provides real-time, non-destructive analysis, and is highly sensitivity to chemical composition changes. It will be demonstrated that by employing this state-of-the-art technique, the precise monitoring of the fractionation process can be ensured, enabling timely adjustments and maximizing the recovery of valuable components from BSG [23].

## 2. Results and Discussion

### 2.1. Characterization of BSG

Table 1 and Table 2 present the results of the analytical parameters determined for BSG.

The moisture content of 83.5% [7] (Table 1) in BSG is indicative of its relatively high water content, a factor that holds implications for storage stability and processing considerations (vide supra). The ash content of BSG is 3.5% in dry matter (dm), according to the literature [7]. Additionally, BSG exhibited a lipid content of 7.3% dm (Table 1) [7]. The total nitrogen analysis revealed a 3.6% dm content, with the calculated protein at 22.6% dm (Table 1). This underscores the significance of proteins as one of the major components of BSG, as supported by the literature [7,24].

The analysis of total free amino acids (TAAs) in BSG was also performed (Table 2). The extraction process facilitated the isolation of a protein hydrolysate predominantly composed of peptides or free amino acids. Note that BSG inherently contains a limited amount of free amino acids, since during the mashing process, these free amino acids migrate into the wort, serving as an important source of nitrogen for the yeast during fermentation. The analysis revealed a concentration of 654 mg/Kg dm. Free amino acids constitute a minor fraction of BSG, accounting for only a small percentage compared to the protein content (22.64% dry matter), indicating the predominant presence of peptides. The essential amino acids present in BSG meet the main requirements outlined by the European Food Safety Authority (EFSA) for amino acids in adults [25]. Furthermore, it is interesting to observe the presence of lysine, typically deficient in cereals, at a level of 62 mg/kg dm in BSG. The high protein content and favorable amino acid profile of BSG enhance the value of this by-product for applications in feed and diverse food products, such as biscuits and flour, or in pasta production [11,26].

**Table 2:** Concentration of free amino acids in brewer’s spent grain (BSG).

Non-Essential AAs	Concentration (mg/kg dm)	Essential AAs	Concentration (mg/kg dm)	Essential AAs for Adults (mg/kg/day) ^1^
Aspartic acid	20 ± 1	Histidine	24 ± 1	10
Glutamic acid	43 ± 1	Threonine	15 ± 0	15
Asparagine	20 ± 0	Methionine	13 ± 1	10.4
Serine	18 ± 0	Tryptophan	14 ± 0	4
Glutamine	70 ± 2	Valine	38 ± 1	26
Alanine	29 ± 1	Phenylalanine	44 ± 1	25
Arginine	125 ± 2	Isoleucine	20 ± 1	20
Glycine	9 ± 0	Leucine	58 ± 1	39
Tyrosine	32 ± 1	Lysine	63 ± 1	30
TOTAL	335 ± 7	TOTAL	320 ± 7	184
TAAs	655 ± 14			

Abbreviations: AAs = amino acids; TAAs = total free amino acids; dm = dry matter. ^1^ Ref. [25].

Next, BSG exhibited starch, residual sugars, and β-glucan contents of 6.7% dm, 2.6% dm, and 0.5% dm, respectively [7,27] (Table 1). The amounts of starch and residual sugars enhance the attractiveness of BSG for industrial purposes, particularly in the production of fermentable sugars [28]. The residual sugars, comprising D-glucose and maltodextrins, persist as remnants from the mashing process post washing and lautering of BSG. Furthermore, the β-glucan content holds promise for diverse applications in functional foods, imparting health benefits [29], and extends to other research areas, such as materials science [30].

The predominant fraction within BSG is composed of total fiber, primarily lignocellulose, amounting to approximately 56.5% dm (Table 1), according to the literature values [7]. This fiber fraction consists of high-value components, i.e., arabinoxylans (that is, hemicellulose), cellulose, and lignin, quantified at 17.1% dm, 19.7% dm, and 19.6% dm, respectively [7] (Table 1). Arabinoxylans, recognized for their versatility, find applications across diverse sectors [11,12,30], particularly in the production of functional food ingredients, owing to their positive impact on human health [31]. For lignin, the soluble fraction comprises 1.4% dm, while the insoluble counterpart constitutes 18.2% dm (Table 1). The total polyphenols (TPs) were quantified at 7.4 mg gallic acid equivalents (GAEs) per gram of dry matter (g^−1^ dm) (Table 3). Additionally, free phenolic acids (FPAs) and bound phenolic acids (BPAs) were determined at 0.8 mg GAE g^−1^ dm and 6.3 mg GAE g^−1^ dm, respectively. The specific value for total polyphenolic acids (TPAs) was 782 µg g^−1^ dm, comprising 152 µg g^−1^ dm of FPAs and 630 µg g^−1^ dm of BPAs. Prominent among the identified FPAs and BPAs were homovanillic (47.5 µg g^−1^ dm) and ferulic acid (247 µg g^−1^ dm), respectively. The presence of polyphenols and phenolic acids underscores BSG’s potential as a feedstock for the production of health-beneficial products, given its known antioxidant, antimicrobial, and anti-inflammatory properties [24].

### 2.2. Fractionation Process of BSG

**Figure 1:**
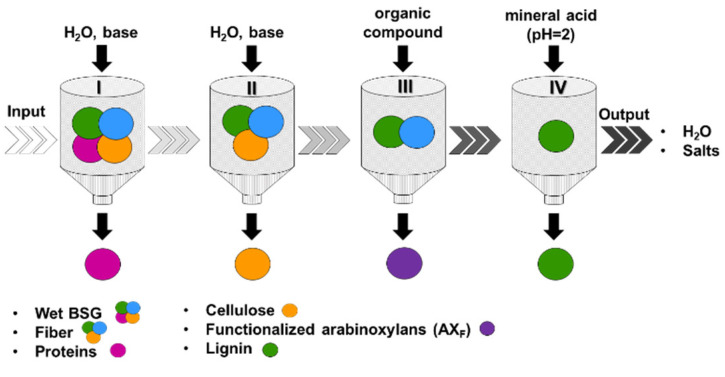
Schematic of the brewer’s spent grain (BSG) fractionation process.

Figure 1 presents a schematic illustration of the sequential four-step BSG fractionation process. Initially, a liquid protein-rich fraction (P) was separated from the solid fiber-rich fraction (F) through treatment with aqueous alkali, preferably NaOH (refer also to Materials and Methods), and the fractions were subsequently collected. The protein separation efficiency, calculated as the percentage of proteins in the liquid fraction relative to the total proteins present in the BSG, was ~63%. This value competes with or even surpasses previously reported yields [14,32]. Various methods have been reported in the literature for protein extraction from BSG [14,15,32]. In the case of alkaline extraction, as employed in this study, the processes achieved protein separation efficiencies ranging from 59 to 77%, demonstrating effectiveness both in performance and in yielding proteins with beneficial functional traits for various applications. However, it is worth noting that under severe conditions, some degradation of amino acids was observed [15]. Ethanolic extraction while achieving protein recoveries in the range of 49–60% requires high temperatures and volatile organic solvents [15]. Finally, enzymatic hydrolysis has been reported to attain protein recovery values of ~80% [15]. However, it is important to note that the industrial-scale use of enzymes might not be economically feasible. This is primarily due to the substantial costs associated with controlling the bioprocesses, which include the need for highly characterized and tightly regulated raw materials and physical parameters.

In the second step, the solid fiber-rich fraction was subjected to treatment in water with an increased alkali loading. This process resulted in the selective separation of the cellulose-rich solid residue (C) from a liquor primarily composed of arabinoxylans and lignin. This resulted in an 84% yield, calculated based on the 19.8% (dry matter) cellulose content within BSG. Next, by adding a carefully chosen organic compound into the liquid stream following cellulose separation, the selective functionalization and concurrent precipitation of the arabinoxylans became feasible. This functionalization represents a strategic dimension, offering the potential for precise adjustment, such as fine tuning, of the compatibility of arabinoxylans when utilized as a filler within a polymer matrix, specifically in the context of composite materials. In a small-scale biorefinery scenario, the revalorization of approximately 5000 tonnes of BSG per year, corresponding to 250,000 hL per year of beer, could yield approximately 4000 tonnes per year of functionalized arabinoxylan-based composite formulations, assuming a minimum filler amount of 4% in the composite formulations.

To illustrate, a benzoic acid congener was added, enabling the selective modification of the polysaccharide through the esterification of hydroxyl groups in the repeating unit. This resulted in altered solubility compared to lignin. The subsequent separation involved precipitating the less soluble arabinoxylan-bound benzoate (AX_F_, Figure 1). Various reaction conditions were tested in this reactive extraction step to optimize performance, including the fiber to water ratio (1:100–1:150 *w*/*v*), fiber to alkali ratio (1:3–1:5 *w*/*w*), benzoic acid congener to arabinoxylans ratio (5–6%, mol/w), temperature (30–50 °C), and time (30 min−1 h). The best conditions (1:150 *w*/*v* solid to liquid ratio, 1:5 *w*/*w* solid to alkali ratio, 30 °C) led to an optimal extraction yield of ~90%. The strategic inclusion of a reactive extraction step in the developed process allows for the direct production of functionalized arabinoxylans, thus significantly enhancing the value of hemicelluloses for biorefineries.

Lastly, lignin (L) could be recovered from the liquor by precipitation at pH ≅ 2–3 (see also Materials and Methods section) with a yield of ~50%. The residual liquid obtained after lignin removal holds potential for dual applications as both a fertilizer and a feedstock to produce biochar, particularly hydrochar [33]. Hydrochar, notably, does not require the drying of the feedstock and offers benefits for a wide range of applications, including biofuel, energy storage, and catalysis [33].

### 2.3. ATR-FTIR Analysis

**Figure 2:**
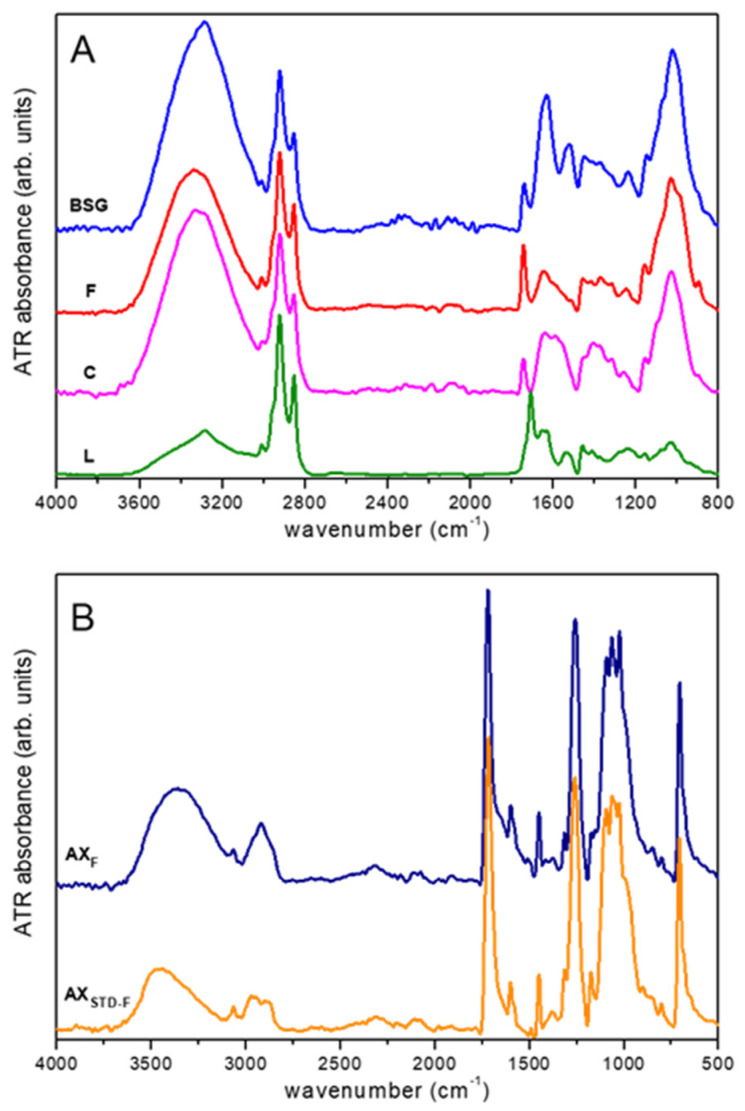
ATR-FTIR spectra: (**A**) BSG, fiber (F), cellulose (C), and lignin (L) fractions; (**B**) functionalized arabinoxylans, AX_F_, obtained using the proposed method, versus an arabinoxylan-bound benzoate synthesized from standard arabinoxylan under the same extraction conditions (AX_STD-F_). Spectra are normalized to the ~2920 cm^−1^ peak (**A**) and ~1720 cm^−1^ peak (**B**) and vertically shifted for clarity.

BSG represents a complex heterogeneous matrix characterized by multiple functional groups typical of lignocellulosic structures. Representative attenuated total reflectance Fourier-transform infrared (ATR-FTIR) spectra of the BSG and various samples resulting from the fractionation process are reported in Figure 2A. The spectrum of the BSG aligns with previous studies [19,34,35], with the main spectral signals assigned based on literature data. The broad band in the 3100–3600 cm^−1^ region is primarily ascribed to the stretching of OH groups of polysaccharides (i.e., cellulose, arabinoxylan) and NH groups of proteins [34,35,36]. A small peak at 3100 cm^−1^ can be related to the stretching of aromatic CH bonds (i.e., lignin) [19], while the signals between 3000 and 2800 cm^−1^ relate to aliphatic CH stretching [35,36,37]. The peak at 1740 cm^−1^ can be attributed to the stretching of C=O groups of hemicellulose, lipids, and/or lignin [19,34,35,37]. The broad band within 1580–1700 cm^−1^ can be mainly related to the amide protein groups (Amide I) and to C=C vibrations of aromatic rings of lignin [34,35,36,38]. Other typical protein signals can be found at ca. 1530 cm^−1^ (Amide II) and 1240 cm^−1^ (Amide III) [19,34,35,36,38]. In addition, signals due to vibrations of conjugated carbonyl groups and aromatic rings (i.e., lignin, ferulic acid) might contribute to the same spectral region (1500–1700 cm^−1^) [19,34,35,36,37,39]. The bands at 1150, 1025, and 895 cm^−1^ can be mainly assigned to vibrations of cellulose and hemicellulose [19,34,35]. As discussed above, the first stage of the BSG fractionation process involves the recovery of the P fraction, which was achieved with a good extraction yield. Consistently, a strong depletion of the amide signals at 1630 cm^−1^, 1520 cm^−1^, and 1240 cm^−1^ can be observed going from the spectrum BSG to that of the F fraction (Figure 2A). In this respect, the ATR-FTIR method might represent an easy route to quickly check the suitability of the separation method. Concerning the separation of cellulose during the second step of BSG fractionation, the spectrum of the C fraction basically shows the same spectral components observed for the F fraction, evidencing variations in the relative signal intensity. This suggests, as expected, that cellulose significantly contributes to the spectrum of the F fraction.

Moreover, the data confirm that the C fraction still contains a certain percentage of other components, as indicated by the presence of the signals at 3100 cm^−1^ and 1740 cm^−1^ assigned to lignin and/or hemicellulose. The peak at 895 cm^−1^ strongly depletes from the F to the C fraction, suggesting that it might be mainly assigned to the CH bending vibrations of hemicellulose [19]. With regard to the spectrum of the L fraction, a strong relative decrease in the intensity of the bands at around 3300 cm^−1^ (i.e., OH stretch) and 1020 cm^−1^, mainly ascribed to cellulose and hemicellulose, can be observed. Moreover, the peak at 3100 cm^−1^ due to the stretching of aromatic CH bonds [19], only marginally present in the spectrum of the C fraction, can be easily detected.

Noticeably, an intense peak at 1710 cm^−1^ can be also observed, which was absent in all of the previous samples. This can probably be assigned to specific carbonyl groups (carboxyl acids) formed upon acidification in the fourth stage of the fractionation process.

The spectrum of the AX_F_ fraction is presented in Figure 2B, alongside the spectrum of a standard arabinoxylan sample that has undergone a similar functionalization process (AX_STD-F_). This comparison is essential for evaluating the formation of ester bonds. The two spectra exhibit remarkable similarity, featuring several distinct peaks (e.g., at 3070 cm^−1^, 1720 cm^−1^, 1260 cm^−1^, and 710 cm^−1^) that were absent in the earlier samples. These peaks can be attributed to the presence of the substituting compound.

In addition to confirming the efficacy of the proposed separation procedure, these findings suggest that the ATR-FTIR technique could be effectively employed as a user-friendly quality control tool for assessing the appropriateness of the extraction process.

## 3. Materials and Methods

### 3.1. Materials

The BSG was collected from the production of a pale ale beer, prepared using Pilsner barley malt, at the brewing pilot plant of the Italian Brewing Research Centre (CERB), University of Perugia (Perugia, Italy). To maintain its integrity, the BSG was stored frozen (−80 °C) in the absence of light until utilization. For analytical purposes, a portion of the BSG was subjected to a drying process in a fluidized bed dryer at 60 °C for 2 h. Following drying, the BSG underwent homogenization and milling to ensure a representative and consistent sample. The resulting homogenized BSG was then securely stored in sealed polyethylene bags under vacuum conditions at room temperature.

All chemicals were purchased from Merck KGaA (Darmstadt, Germany) and used without further purification, unless otherwise noted.

### 3.2. Analytical Methods

The composition of BSG was determined using the following set of parameters: moisture content, total nitrogen, protein content, β-glucans, starch, residual sugars, arabinoxylans, cellulose, lignin, lipid content, ash content, total polyphenols, phenolic acids, and total free amino acids. The analyses were performed according to Analytica EBC methods (A-EBC) and Official Methods of Analysis (AOAC), unless otherwise noted.

#### 3.2.1. Determination of Moisture, Lipid, Ash, Total Nitrogen, and Protein Content

The moisture, lipid, and ash content were analyzed following A-EBC 12.2 [40], 6.10 [41], and AOAC 14.006 [42] methods, respectively.

The assessment of moisture content in BSG involves conducting a loss-of-mass analysis through drying under specific conditions. Fatty substances present in the ground cereals are extracted using petroleum ether in a Soxhlet-type extraction apparatus. Following extraction, the solvent is evaporated, and the remaining fatty residue is weighed.

For ash determination, a defined quantity of the sample is placed in a preheated platinum capsule. Subsequently, the capsule undergoes incineration in a muffle furnace at 550 °C for approximately 4 h until the sample achieves a light gray or white coloration. After cooling within a desiccator, the capsule is re-weighed to ascertain the ash content.

Total nitrogen analysis was performed using the A-EBC 4.3.1 method (Kjeldahl method) [43]. The nitrogenous compounds present in BSG undergo digestion with hot H_2_SO_4_ in the presence of a catalyst, resulting in the formation of ammonium sulfate. The digestate is then made alkaline using a NaOH solution, inducing the release of ammonia, which is then distilled into an excess of boric acid solution. Subsequently, the ammonia is titrated with a standard acid solution. This analytical process was carried out using a Kjeldahl digestion rack (FOSS Analytics, Hillerød, Denmark) and a Kjeltec^TM^ 9 Distillator (FOSS Analytics, Denmark). The protein content was obtained by multiplying the total nitrogen value by the specific nitrogen conversion factor for barley malt, which is 6.25.

#### 3.2.2. Determination of β-Glucans, Starch, and Residual Sugars

The quantification of β-glucans was performed using the β-Glucan Assay Kit (Megazyme International, Wicklow, Ireland), in accordance with the method A-EBC 4.16.1 [44]. To determine the starch content, two different methodologies were adopted. In the first approach, the Total Starch Assay Kit (Megazyme International, Ireland) was used. This involved the removal of D-glucose and maltodextrins from the BSG through alcohol washing. In the second method, the starch content was assessed without subjecting the BSG to washing. Using this procedure, the quantification encompassed both the starch content and residual sugars originating from the mashing process. The quantification of residual sugars was achieved by determining the difference between the results obtained from the two methods.

#### 3.2.3. Determination of Arabinoxylans, Lignin, and Cellulose

The arabinoxylan (AX) content was determined according to the method described by Marconi et al., 2020 [45]. The quantification of lignin content followed the standard procedure outlined by the National Renewable Energy Laboratory (NREL) [46]. In the determination of cellulose content in the BSG, a calculation by difference methodology was applied. This method entailed subtracting the measured values of various major components of the BSG, including ash, lipids, protein, starch, residual sugars, β-glucans, AX, and lignin, from the total weight of the sample. The resultant value represented the remaining weight, attributed specifically to the cellulose content in BSG.

#### 3.2.4. Determination of Total Polyphenols, Phenolic Acids, and Free Amino Acids

The total polyphenol (TP) content of the BSG, including both free (FP) and bound (BP) fractions, was quantified using the Folin–Ciocalteu spectrophotometric method [47]. The individual content of free phenolic acids (FPA) and bound phenolic acids (BPA) in the BSG was determined following the method outlined by Stagnari et al. [48]. The total free amino acid (TAA) content in the BSG was measured based on the methodology reported by Marconi et al. [49].

### 3.3. Fractionation of BSG

The fractionation of BSG was carried out following a patented protocol [23]. The process utilized wet BSG as the feedstock. Initially, the BSG was mixed with water and a pre-determined amount of alkali, at temperatures ranging from 50 °C to 70 °C. The resulting slurry was subjected to stirring for at least 2 h, followed by cooling, allowing the separation of the liquid protein-rich fraction from the solid fiber-rich fraction through filtration. The yield was calculated based on the total nitrogen in the liquid phase and the total nitrogen in the initial dried matter. Subsequently, the fibrous fraction underwent extraction with an alkali solution in water for a minimum of 6 h, while being stirred at temperatures between 30 and 50 °C. After cooling and filtration, a solid residue rich in cellulose and a liquid fraction containing primarily arabinoxylans and lignin were collected. The yield of cellulose was calculated based on the weight of the dried solid product and the amount of cellulose in the initial dried matter. Next, the liquid fraction was kept under stirring (at T = 30–50 °C) and treated with a benzoic acid congener (1–9% m/V). This process resulted in the selective separation of a solid arabinoxylan-bound benzoate (AXF), which was collected via centrifugation after filtration. The yield of arabinoxylan-bound benzoate was determined based on the amount of free arabinoxylans remaining in the mother liquor and the arabinoxylans in the starting dried matter. Finally, solid lignin was recovered from the remaining mother liquor by carefully adjusting the pH to ~2–3. The yield of lignin was calculated based on the weight of the dried solid product and the amount of lignin in the initial dried matter.

### 3.4. ATR-FTIR Spectroscopy

Fourier-transform infrared (FTIR) spectra in the attenuated total reflection (ATR) configuration were obtained using a compact FTIR spectrometer (mod. Alpha, Bruker Optics, Ettlingen, Germany) equipped with an ATR module (mod. Platinum, Bruker Optics, Germany) involving a single reflection diamond crystal. The OPUS 7.5 Bruker Optics software was employed for the acquisition and analysis of the IR spectra. These were recorded in the 400–5000 cm^−1^ range averaging over 30 scans, with a resolution of 2 cm^−1^. The spectra were corrected using the “atmospheric compensation” and baseline routines implemented in the OPUS 7.5 program.

## 4. Conclusions

This study highlights the effectiveness of a novel sequential extraction process for wet brewer’s spent grain (BSG), yielding valuable components for various industries. BSG, in its wet form, reduces economic and energy costs, offering a sustainable alternative to drying pre-treatment. The fractionation process achieves high extraction yields for high-value biopolymers, i.e., proteins (~65%), cellulose (~85%), arabinoxylans (~90%), and lignin (~50%), under mild alkaline conditions, using water as a green solvent. Notably, it is crucial in several areas [50,51] to utilize green solvents, as their use is pivotal in addressing a wide range of sustainability goals.

Incorporating a reactive extraction step into the developed process strategically enables the direct and efficient production of functionalized arabinoxylans, thereby substantially amplifying the value of hemicelluloses for biorefineries [52,53].

Furthermore, this study effectively showcased the utility of ATR-FTIR spectroscopy for monitoring and ensuring the reproducibility of the BSG fractionation process. Notably, the observed spectral changes provided concrete confirmation of successful protein extraction and served as validation for the proposed arabinoxylan extraction procedure. In summary, ATR-FTIR spectroscopy proves to be a user-friendly tool for conveniently assessing the suitability of the extraction process, demanding minimal sample preparation.

Overall, this study underscores the substantial potential of the sequential extraction process for BSG in accessing high-value biopolymers. The process charts a course toward a zero-waste paradigm in breweries and facilitates the market-competitive utilization of BSG-derived components, with a strong emphasis on sustainability.

## 5. Patents

Title: Process for treating of brewing industry by-products.

Inventors: Ombretta Marconi, Assunta Marrocchi.

Patent Number: WO2023012841 (A1) Date of Issue: 9 February 2023.

This manuscript reports work that resulted in a patented invention titled “Process for treating of brewing industry by-products” [23]. The patent was issued on 9 February 2023 with WO2023012841 (A1). The invention, briefly described as a process for treating wet brewer’s spent grains for the extraction and integral recovery of their main homogeneous components, specifically protein, cellulose, hemicellulose, and lignin, is directly related to the research presented in this manuscript and has significant implications in the valorization of food waste.

## Figures and Tables

**Table 1:** Determination of BSG composition ^1^.

BSG Components	Concentration (% dm ^2^)
Moisture	83.5 ± 0.2 ^3^
Total nitrogen	3.62 ± 0.02
Proteins	22.64 ± 0.12
Lipids	7.33 ± 0.23
Ash	3.51 ± 0.02
Starch	6.8 ± 0.9
Residual sugars	2.7 ± 0.0
Total fiber	56.54 ± 1.3
β-glucans	0.54 ± 0.07
Cellulose	19.78 ± 0.82
Arabinoxylans	17.11 ± 1.86
Total lignin	19.65 ± 0.25
Soluble lignin	1.42 ± 0.07
Insoluble lignin	18.23 ± 0.32

^1^ Values are the average of duplicate ± standard deviation; ^2^ dry matter; ^3^ %.

**Table 3:** Polyphenol and phenolic acid analysis in BSG.

Polyphenol	Concentration (mg GAE g^−1^ dm)
FP	0.84 ± 0.03
BP	6.35 ± 0.34
TOTAL	7.41 ± 0.12
**Free Phenolic Acids**	**Concentration (µg g^−1^ dm)**
α-resorcylic acid	17.1 ± 2.2
Syringic acid	24.7 ± 2.7
Homovanillic acid	47.5 ± 3.1
p-Coumaric acid	10.0 ± 0.6
Salicylic acid	19.8 ± 0.7
Ferulic acid	21.1 ± 1.4
Sinapic acid	11.7 ± 0,9
TOTAL	151.9 ± 7.8
**Bound Phenolic Acids**	**Concentration (µg g^−1^ dm)**
α-resorcylic acid	13.9 ± 1.8
p-hydroxybenzoic acid	4.7 ± 0.6
Vanillic acid	11.9 ± 0.3
Caffeic acid	27.3 ± 1.4
p-coumaric acid	170.9 ± 6.7
Salicylic acid	126.1 ± 4.1
Ferulic acid	247.1 ± 6.4
Sinapic acid	42.2 ± 3.0
TOTAL	630.1 ± 93.4
Total phenolic acids	782.0 ± 5.8

Abbreviations: FP = free polyphenols; BP = bound polyphenols; dm = dry matter.

## Data Availability

The data presented in this study is available on request from the corresponding author. However, the data is not publicly available because the process for extracting BSG is patented.
